# Genetic Analysis and Fine Mapping of QTL for the Erect Leaf in Mutant *mths29* Induced through Fast Neutron in Wheat

**DOI:** 10.3390/biology13060430

**Published:** 2024-06-11

**Authors:** Zhixin Yang, Jiayu Gu, Minghui Zhao, Xiaofeng Fan, Huijun Guo, Yongdun Xie, Jinfeng Zhang, Hongchun Xiong, Linshu Zhao, Shirong Zhao, Yuping Ding, Fuquan Kong, Li Sui, Le Xu, Luxiang Liu

**Affiliations:** 1 College of Agriculture, Yangtze University, Jingzhou 434023, China; yangzx0129@163.com (Z.Y.); 15179718627@163.com (X.F.); 501140@yangtzeu.edu.cn (L.X.); 2 State Key Laboratory of Crop Gene Resources and Breeding, National Engineering Laboratory for Crop Molecular Breeding, National Center of Space Mutagenesis for Crop Improvement, CAEA Research and Development Center on Nuclear Technology Applications for Irradiation Mutation Breeding, Institute of Crop Sciences, Chinese Academy of Agricultural Sciences, Beijing 100193, China; gujiayu@caas.cn (J.G.); guohuijun@caas.cn (H.G.); xieyongdun@caas.cn (Y.X.); xionghongchun@caas.cn (H.X.); zhaolinshu@caas.cn (L.Z.); zhaoshirong@caas.cn (S.Z.); dingyuping@caas.cn (Y.D.); 3 Dry-Land Farming Institute of Hebei Academy of Agricultural and Forestry Sciences, Hengshui 053000, China; 4 China Institute of Atomic Energy, Beijing 102413, China; fqkong@ciae.ac.cn (F.K.); lisui@ciae.ac.cn (L.S.)

**Keywords:** wheat, erect leaf, fine mapping, transcriptome analysis

## Abstract

**Simple Summary:**

Erect leaves are one of the important phenotypes for plants to adapt to dense planting. This study obtained the erect leaf mutant *mths29* through fast neutron irradiation and directional breeding. Dynamic observation of lamina joint development in the mutant and its genetic parent Heng S29 revealed an extreme phenotype during the booting stage, characterized by the complete absence of lamina joint on the inverted second leaves and flag leaves, resulting in a close adhesion of the leaf blade to the stem and the formation of an erect leaf phenotype. Through map-based cloning, the erect leaf QTL was localized within a physical interval of 1.03 Mb on chromosome 5A, and four potential candidate genes were predicted. Here, we demonstrate that *mths29* represents a novel genetic resource for erect leaf traits in wheat. This study contributes to a better understanding of lamina joint development in graminaceous and aids in shaping plant architecture for denser planting.

**Abstract:**

The erect leaf plays a crucial role in determining plant architecture, with its growth and development regulated by genetic factors. However, there has been a lack of comprehensive studies on the regulatory mechanisms governing wheat lamina joint development, thus failing to meet current breeding demands. In this study, a wheat erect leaf mutant, *mths29*, induced via fast neutron mutagenesis, was utilized for QTL fine mapping and investigation of lamina joint development. Genetic analysis of segregating populations derived from *mths29* and Jimai22 revealed that the erect leaf trait was controlled by a dominant single gene. Using BSR sequencing and map-based cloning techniques, the QTL responsible for the erect leaf trait was mapped to a 1.03 Mb physical region on chromosome 5A. Transcriptome analysis highlighted differential expression of genes associated with cell division and proliferation, as well as several crucial transcription factors and kinases implicated in lamina joint development, particularly in the boundary cells of the preligule zone in *mths29*. These findings establish a solid foundation for understanding lamina joint development and hold promise for potential improvements in wheat plant architecture.

## 1. Introduction

The structure of plants directly influences the aboveground biomass, population structure, and final yield formation of crops, which are crucial for crop growth and yield [1,2]. To meet the increasing demand of the ever-growing population, developing compact plant architecture suitable for dense planting is a key factor in increasing wheat yield [3]. Studies have shown that the erect canopy structure of crops can enhance ventilation and stress resistance within dense canopies, allowing more light to penetrate the upright upper leaves and reach the lower leaves, thereby improving the radiation use efficiency (RUE) and increasing yield [4,5]. Consequently, there is growing attention to erect leaf plant types in the current breeding process [6,7,8].

The lamina joint, comprising the collar, ligule, and auricle, serves as a critical determinant of leaf erectness in agricultural studies. The collar offers essential mechanical support for precise leaf angle adjustment [9]. Simultaneously, the ligule, characterized by its transparent, tongue-like structure, acts as a protective barrier, safeguarding the emerging leaf from potential harm. Additionally, the auricle, positioned on either side of the lamina joint, serves to secure the connection between the leaf sheath and the main stem, preventing detachment [10]. Regular cellular development serves as the fundamental basis for the formation of the lamina joint. Research has revealed that during the initial stages of tissue differentiation, the preligule zone emerges at the junction between the leaf blade and the leaf sheath. Subsequently, the mature lamina joint is established through the ongoing proliferation and differentiation of cells [11]. The aberration in the lamina joint structure typically involves the inhibition of longitudinal cell elongation in the adaxial region (proximal to the leaf primordium) and an increase in the division of sclerotic cells in the abaxial region (distant from the leaf primordium), consequently resulting in the formation of erect leaves [12,13].

Numerous erect leaf genes have been identified in maize and rice, including *lg1* [14], *lg2* [15], *rs2* [16], *Rs1* [17], *Lg3* [18], *Kn1* [19], and *Gn1* [20]. Among them, *liguleless1* (*LG1*) and *liguleless2* (*LG2*) are extensively studied genes within the Poaceae. Towards the end of the 20th century, the *lg1-R* mutant of maize exhibited a complete absence of the early lamina joint, with only a small ligule appearing in later stages. *ZmLG1*, the gene responsible for the erect leaf trait in this mutant, encodes a nuclear localization protein containing the SBP domain. It engages in brassinosteroid (BR) and auxin signal transduction via pathways such as DRL1/2-LG1-RAVL1, ILI1-LG1, KN1-LG2-LG1-ARF, and TCP-LG1-ARF [21,22,23]. Conversely, at the *lg2-R* mutant, the lamina joint is absent at the midvein of the first three leaves, while residual leaf auricles are detected at leaf margins. Later stages exhibit displaced ligules and auricles. *ZmLG2*, governing erect leaf traits, encodes a bZIP protein and is situated upstream of *ZmLG1*, determining the lamina joint’s developmental position and initiating initial tissue differentiation [24,25]. Additionally, the ectopic expression of KNOX protein in the lamina joint alters the polarity distribution of boundary cells crucial for lamina joint formation, shifting it from longitudinal to transverse orientation. This disruption in auxin transmission leads to plant dwarfism, lamina joint cell disintegration, and irregularities in leaf morphology [26].

*TaSPL8* stands as the wheat homolog of *ZmLG1*, is the sole cloned gene associated with erect leaf traits in wheat, currently. It modulates the expression of cell elongation-related genes via the plant hormone pathway, ultimately leading to the development of erect leaves [27]. Additionally, in the erect leaf mutant *LM* from *Aegilops tauschii*, the candidate gene *Lgt* was genetically mapped to chromosome 5DS [28]. Seventeen quantitative trait loci (QTLs) linked to leaf angle were identified within 269 recombinant inbred lines derived from Yanda1817 and Beinong6 [29]. Furthermore, three major leaf angle QTLs were detected in recombinant inbred lines (RILs) developed from ND3331 and Zang1817 [30]. These findings underscore the multifaceted influence of factors such as genetic background and plant hormones on the development of the lamina joint. Therefore, continuous exploration of the mechanism underlying wheat erect leaf formation holds significant agricultural significance. 

The wheat erect leaf mutant *mths29*, induced via radiation mutagenesis, was a favorable donor for plant architecture investigation. the mutant displayed a complete absence of lamina joint in the inverted second leaf and flag leaf, resulting in leaves positioned closely to the stem, a notable reduction in leaf angle, and compact plant architecture, implying potential advantages for increasing yield in densely planting conditions. The objectives of this study are to (1) investigate the developmental characteristics of lamina joint in *mths29*; (2) fine map the QTL region using derived segregation populations; (3) investigate the mechanisms underlying erect leaves through transcriptome analysis; (4) identify candidate genes by comparative and expression analysis; (5) develop molecular markers that can assist to select compact plant architecture in future breed program.

## 2. Materials and Methods

### 2.1. Plant Materials

Ripe and plump dry seeds of winter wheat cultivar Heng S29 were subjected to fast neutron irradiation at the China Institute of Atomic Energy (Beijing, China), with irradiation energy set at 14 MeV and irradiation dose at 14 Gy. The M_1_ to M_4_ generations had been subjected to single-seed sowing, with two rows planted per plant line and a plant spacing of 20 cm. Untreated seeds of Heng S29 were served as the control, and mutant single plants had been selectively bred. The stable homozygous erect-leaf single plants from the M_4_ generation were designated *mths29*. Subsequently, *mths29* was crossed with the elite winter wheat cultivar Jimai22 (with normal leaves) to generate a segregating population for genetic mapping of the erect leaf QTL. Using *mths29* as the maternal parent and Jimai22 as the paternal parent, F_1_ was formed through hybridization, segregation population was formed through F_1_ self-crossing. Each F_2_ plant was self-pollinated to generate F_2:3_ families. Employing marker-assisted breeding, 21 heterozygous individuals exhibiting recombination were selected from the F_2:3_ generation and advanced to the F_4_ generation as single plant-derived families. Utilizing molecular markers closely linked to the erect leaf phenotype, 27 key heterozygous recombinants were identified from the F_4_ generation and self-pollinated to the F_5_ generation.

Subsequently, to elucidate the mechanism underlying the formation of erect leaves in *mths29* through transcriptomic analysis, Jimai22 was used as the recurrent parent to backcross with the F_1_ population of Jimai22 × *mths29* for three rounds, resulting in the BC_3_F_1_ generation. The resulting population was then self-pollinated for three generations, and in the BC_3_F_3_ generation, individuals exhibiting the normal leaf phenotype were designated as LG-JM22, while those displaying the erect leaf phenotype were designated as LG-mt. The aforementioned populations and their parental lines were uniformly grown at the Bei Field Station of the Institute of Crop Sciences, Chinese Academy of Agricultural Sciences (Beijing), with 15 plants planted within every 2 m row for optimal field management.

### 2.2. RNA Extraction and Quality Determination

Total RNA was extracted from the booting stage of plants exhibiting erect leaves and normal leaves utilizing TransZol (TransGen Biotech, Beijing, China). The RNA extraction was performed by Biomarker Technologies company for subsequent experimentation. The concentration of RNA was determined using a NanoDrop One spectrophotometer (Thermo Scientific, Waltham, MA, USA).

### 2.3. Bulked Segregant RNA-seq (BSR) of F_2_

Based on lamina joint morphology, two extreme bulks were constructed within the F_2_ population, each comprised of 30 individuals with normal leaves (T13) and 30 individuals with erect leaves (T14), respectively, for bulked segregant analysis (BSR) aimed at preliminary mapping of erect leaf QTL. Following RNA extraction, individual cDNA libraries were constructed for each bulk, which were subsequently subjected to sequencing on the Illumina HiSeqTM platform. Utilizing filtered clean reads, sequence alignment was performed against the latest Chinese Spring reference genome released by the International Wheat Genome Sequencing Consortium (IWGSC) using STAR_2.7.6a software. Single nucleotide polymorphism (SNP) detection and annotation were conducted using GATK and SnpEff. Association analysis between SNPs and erect leaf formation was performed using ED^5^ and SNP-index [23,24].

### 2.4. Resequencing Analysis

Leaves from 10 individual plants of *mths29* and Jimai22, respectively, were collected to construct parental pools. The DNA from each parental individual was pooled together to form the parental mixed pool. These pools were then submitted to Annuo Youda Gene Technology (Beijing, China) Co., Ltd. for whole-genome resequencing. High-confidence variant sites were identified and filtered using GATK-4.2.1.0 software.

### 2.5. DNA Extraction and Determination of DNA Quality

DNA extraction from individual plants in the population was conducted using the PVP 40 method [26]. The integrity and quality of DNA were assessed through 1% agarose gel electrophoresis with GelRed staining. DNA concentration was quantified using a NanoDrop One spectrophotometer (Thermo Scientific, USA). Subsequently, DNA samples were diluted to a concentration of 150 ng/μL and stored at −80 °C.

### 2.6. Development of Molecular Markers for Fine Mapping 

Utilizing BSR and resequencing data, high-quality SNP loci between Jimai22 and *mths29* were screened. Specific primers were designed using the IWGSC website (http://www.wheatgenome.org/) and Polymarker (http://polymarker.tgac.ac.uk/). Each of the two allele-specific forward primers was labeled with a FAM (5′-GAAGGTGACCAAGTTCATGCT-3′) or a HEX (5′-GAAGGTCGGAGTCAACGGATT-3′) tail at the 3′ end. KASP markers were employed for genotyping individual plants from the F_2_ to F_5_ populations. Combined with field phenotype data, recombinant individuals were identified to progressively narrow down the candidate intervals for erect leaf QTLs.

When SNPs cannot be developed into KASP primers, specific primers are designed within a 500 bp interval upstream and downstream of the target SNP based on the Chinese Spring reference genome. These primers are used for PCR amplification in Jimai22, *mths29*, and recombinant individuals. By aligning the sequences with those of the parental lines, the genotypes of the recombinant individuals in the F_5_ generation are determined. Combined with field phenotype data and molecular markers, this approach facilitates further narrowing down the target interval for QTL localization.

### 2.7. Transcriptome Assembly of BC_3_F_3_

During at booting stage, lamina joints of the inverted second leaves were collected from LG-JM22 and LG-mt in the BC_3_F_3_ generation of Jimai22 and *mths29*. Ten individual lamina joint samples were collected and pooled into single samples, with each sample having three biological replicates, resulting in a total of six samples. These samples were subjected to transcriptome analysis by Biomarker Technologies company. Differential expression genes (DEGs) were identified based on a 1.5-fold change in expression levels and a false discovery rate (FDR) less than 0.05, aiming to elucidate the mechanism underlying the formation of erect leaves in *mths29*.

### 2.8. Reverse Transcription and Quantitative Real-Time PCR Analyses

We employed the PrimeScript RT Reagent Kit with gDNA Eraser (TaKaRa, Beijing, China) for first-strand cDNA synthesis, ensuring genomic DNA removal. Quantitative real-time PCR was carried out using PerfectStart Green qPCR SuperMix (TransGen Biotech, Beijing, China) on the CFX 96 Real-Time System (Bio-Rad, Hercules, CA, USA), following the manufacturer’s instructions. ACTIN was utilized as an endogenous control for expression normalization, and each sample was subjected to at least 3 technical replicates (Table S1). Relative expression levels were determined using the 2^−ΔΔCT^ method [27].

## 3. Results

### 3.1. Phenotypic Analysis of Wheat Erect Leaf Mutant mths29

**Figure 1:**
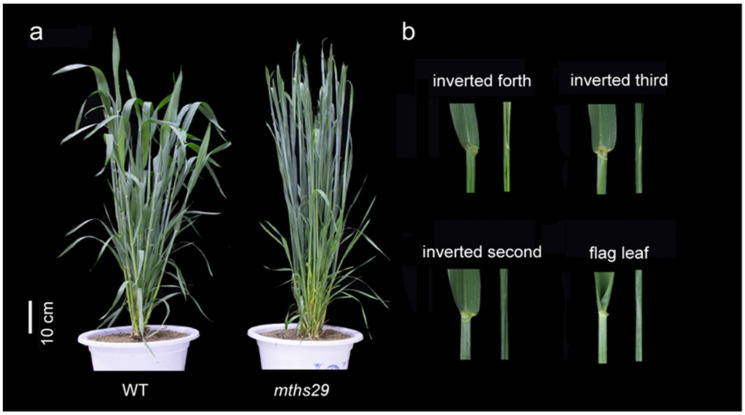
Comparative Analysis of WT and *mths29* phenotypes. (**a**) Comparative depiction of the overall plant morphology between WT and *mths29*. Scale: 10 cm. (**b**) Detailed photographs of the lamina joint characteristics in the inverted second leaves, third leaves, fourth leaves, and flag leaves of WT and *mths29* taken during the booting stage.

We identified an erect leaf mutant, *mths29*, which exhibited a significantly reduced leaf angle and a compact plant architecture compared to the wild type, HengS29 (WT) (Figure 1a). During the regreening stage, the basal leaves of the *mths29* mutant exhibited normal development, showing no significant deviation in plant architecture compared to WT. However. at the heading stage, the *mths29* mutant exhibited a deficiency in lamina joint development in the uppermost leaves, with severity increasing as leaf position ascended. Specifically, the ligule and collar tissue of the inverted fourth leaf degenerated, while the auricle structure remained intact. The shape of the inverted third auricle degraded, leaving behind white protrusions. The lamina joint structure of the inverted second leaf and flag leaf was absent, resulting in excessively smooth sheaths and leaves, and a leaf angle close to 0°. By the mature stage, the plant architecture was compact, with leaves erect and positioned closely to the stem in *mths29* (Figure 1b).

Among the 1764 individuals in the F_2_ population, 1310 exhibited the erect leaf phenotype, while 454 displayed a normal plant phenotype. A chi-square goodness-of-fit test demonstrated that the occurrence of the erect-leaf phenotype adheres to a 3:1 segregation ratio (χ^2^ = 0.51 < χ^2(0.05, 1)^ = 3.84, *p* = 0.47 > 0.05). This suggests that a single dominant gene governs the erect-leaf trait in *mths29* (Table 1). 

### 3.2. Gene Mapping by BSR-seq Assay

**Figure 2:**
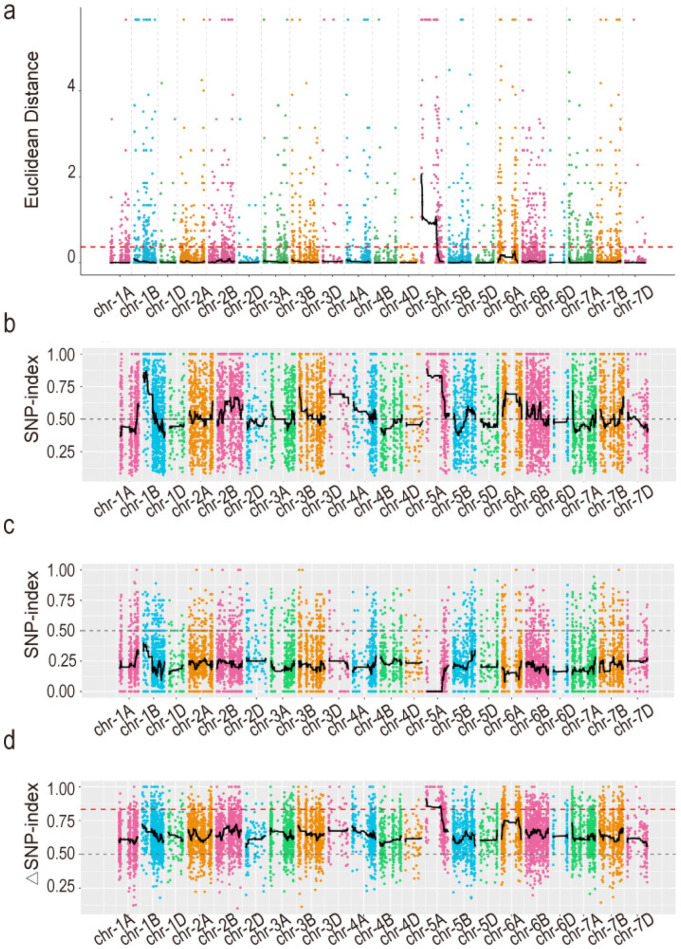
Preliminary mapping results of erect leaf gene. (**a**) Results of the ED^5^ correlation analysis. The black line represents the fitted ED value, while the red dotted line denotes the significant correlation threshold. (**b**–**d**) Results of SNP-index association analysis. (**b**) SNP-index distribution of the erect leaf mixing pool, (**c**) SNP-index distribution of the ordinary leaf mixing pool, and (**d**) ΔSNP-index distribution. The red line signifies the threshold with a confidence level of 0.90, and the gray line represents the threshold with a confidence level of 0.50.

To initially map the erect leaf QTL associated with the lamina joint trait in *mths29*, we conducted bulked-segregant RNA sequencing (BSR-seq) using extreme phenotypes in the F_2_ population. The resulting T13 and T14 generated 37.62 and 46.86 Gb of clean reads, respectively. These clean reads were aligned to the reference genome Chinese Spring v2.1 [28], identifying 139,846 single nucleotide polymorphisms (SNPs). After filtering, 9150 high-quality SNP loci were obtained. In the association analysis using ED5, with a correlation threshold of 0.37, one associated region was identified as located on chromosome 5A, spanning a length of 504.28 Mb and encompassing 3717 genes, among which 48 genes contained non-synonymous mutation SNP loci. In the SNP-index association analysis, SNP-index association analysis was conducted on the two extreme bulks, yielding ΔSNP-index values after fitting. The correlation threshold was set at 0.667, resulting in the identification of one associated region on chromosome 5A with a region length of 478.62 Mb, containing 3359 genes, of which 39 genes harbored non-synonymous mutation SNP loci. Taking the intersection of the two association analysis results, the erect leaf QTL was preliminarily localized on chromosome 5A, with an associated region spanning 478.62 Mb and containing 3359 genes (Figure 2a,b).

### 3.3. Fine Mapping of mths29 Erect Leaf QTL

**Figure 3:**
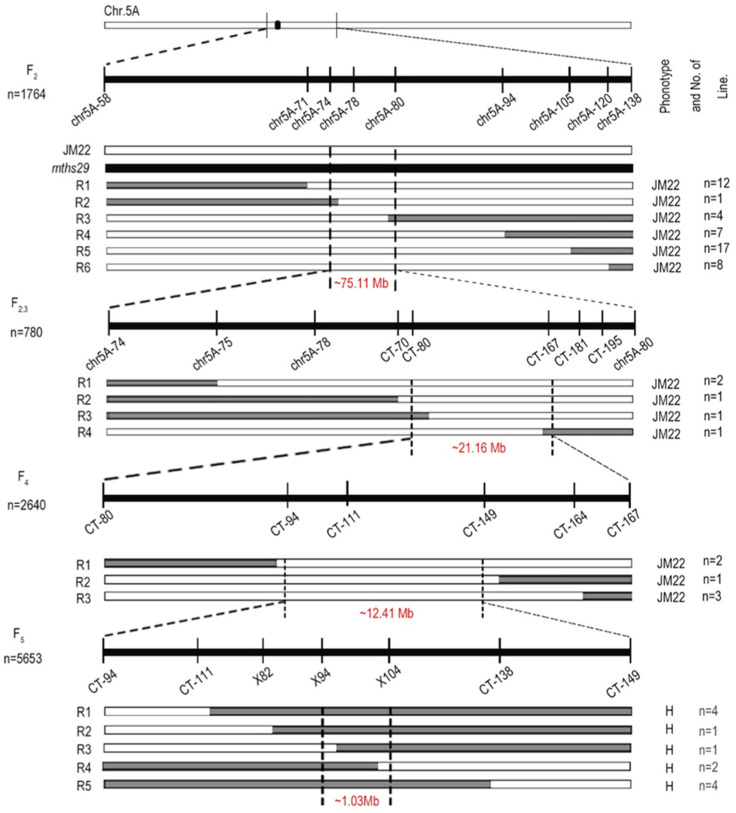
Fine mapping of the lamina joint gene using key recombinant events. Mapping was conducted employing markers developed from polymorphisms within the expressed genes. The left side represents the corresponding recombined individual plant type in this population, and the Arabic numerals on the right indicate the individual plant number of respective type recombinants. The dotted line delineates the physical interval ascertained through fine mapping.

A total of 575 SNPs located on chromosome 5A were screened, and 282 high-quality SNPs were identified, which can be used to develop molecular markers. Initially, employing an F_2_ population comprising 1764 individual plants, the erect leaf QTL was localized within the physical interval of 75.11 Mb on chromosome 5A. Subsequently, leveraging 780 recombinant plants from the F_2:3_ generation, the QTL was further pinpointed to a 21.16 Mb interval spanning from 291.28 Mb to 312.44 Mb on chromosome 5A. A heterozygous plant from the previous generation was utilized to construct the F_4_ generation, comprising 2640 individual plants, leading to the refinement of the erect leaf QTL’s localization to the interval of 295.78–308.19 Mb, spanning approximately 12.41 Mb. Finally, employing a population expansion in the F_5_ generation consisting of 5653 individual plants, and adding more molecular markers, the erect leaf QTL was precisely mapped between markers *X94* and *X104*. This mapping corresponds to the genomic interval of 299.87–300.90 Mb on chromosome 5A, with a physical distance of 1.03 Mb (Figure 3).

### 3.4. Candidate Genes Analysis

**Figure 4:**
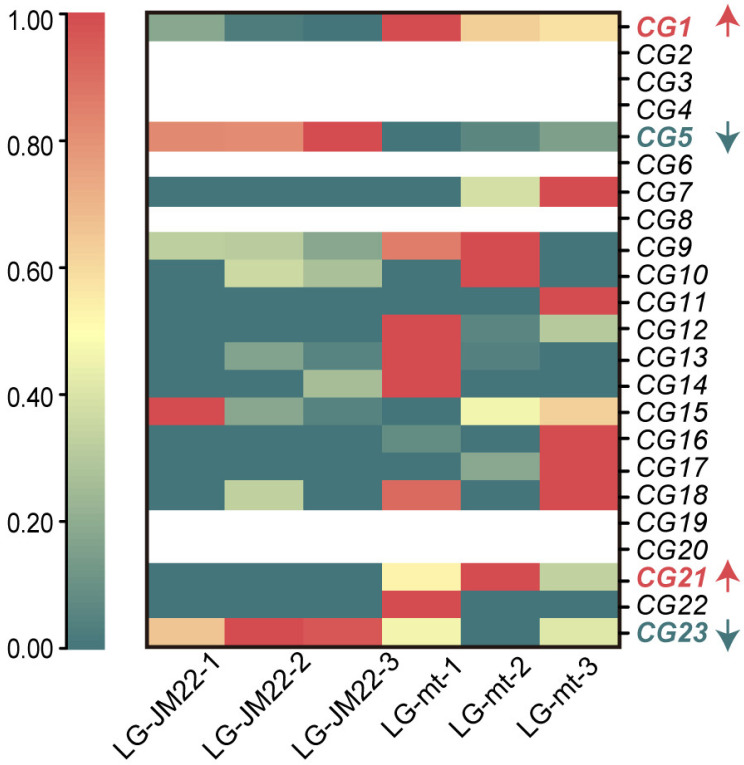
Transcriptome differential expression of candidate genes as ungraded log_2_ expression level. Label candidate genes with different colors. Red and red arrows indicate candidate genes that are upregulated, and green and green arrows indicate candidate genes that are downregulated.

According to the genome of Chinese Spring, 23 annotated genes within the mapped region are considered potential candidate genes (CG) for *mths29*. These genes are designated as *CG1*-*CG23*, comprising 9 high-confidence genes and 14 low-confidence genes. The coding sequences (CDS) of the high-confidence genes were sequenced, revealing no variation sites between LG-JM22 and LG-mt. The expression profiles of these 23 genes were analyzed in the transcriptome of LG-JM22 and LG-mt at the booting stage. It was observed that 7 genes exhibited no expression during this period, 11 genes were expressed at low levels with no significant difference between the two conditions, and 1 gene showed high expression without significant differential expression. Consequently, these genes are less likely to be candidate genes for the erect leaf trait. Among the remaining four candidate genes (*CG1*, *CG5*, *CG21*, and *CG23*), *CG1* (FC = 1.56, FDR = 0.006) and *CG21* (FC = 1.67, FDR = 0.024) exhibited significant upregulation, while *CG5* (FC = −1.56, FDR = 0.002) and *CG23* (FC = −2.17, FDR = 0.018) showed significant downregulation, indicating their potential candidacy for the erect leaf trait (Figure 4).

### 3.5. The Change in Cells Related to Lamina Joint Development for Transcriptome

**Figure 5:**
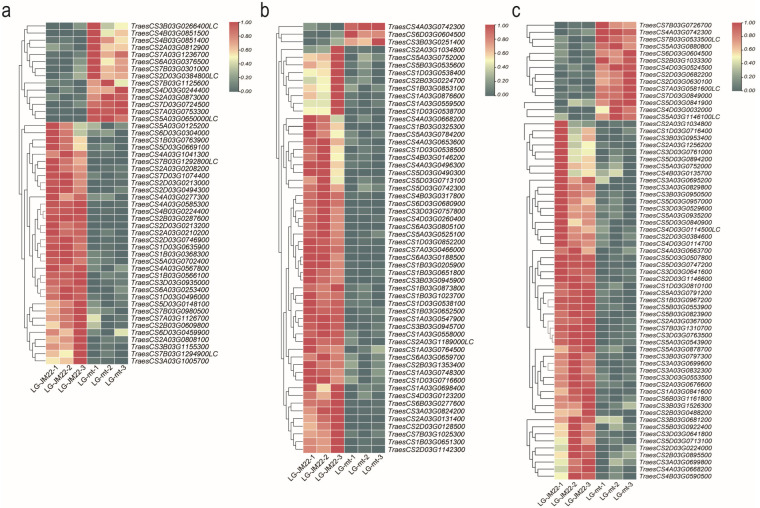
Expression profiles of reported genes related to lamina joint development in the transcriptome. The heat maps depict the expression profiles of genes associated with boundary genes (**a**), cell expansion (**b**), and cell proliferation (**c**) of the lamina joint and adjacent leaf parts, presented as unscaled log2 expression levels.

The ordered cellular structure is fundamental to the normal development of the lamina joint. Aberrations in cell composition, division, and proliferation can result in morphological variations in the lamina joint. Differential expression analysis of LG-JM22 and LG-mt revealed involvement of several gene families in the formation of the preligule zone: 1 gene from the Knotted1-like homeobox family (KNOX), 1 gene from the WUSCHEL HOMEOBOX family (WOX), 29 genes from the No apical meristem family (NAC), and 9 genes from the Squamosa promoter binding protein-like family (SBP). Additionally, a majority of the Asymmetric leaves homologous genes (AS1/AS2), totaling eight genes, exhibited a downward expression trend (Figure 5a). Moreover, the expression levels of genes involved in cell division displayed a decreasing trend in LG-mt. These include 3 genes from the DWARF family, 4 genes from the WARC family, 11 genes encoding cell cycle cyclins proteins, 20 genes encoding expansin proteins, and 11 genes associated with glycosylation (Figure 5b). Genes related to cell proliferation, such as 8 glutamate carboxypeptidase genes and 27 cysteine protease genes, were also downregulated in the majority of LG-mt (Figure 5c). Verification by qPCR confirmed the expression patterns of eight of these genes, consistent with the transcriptome data (Supplement Figure S1).

### 3.6. Transcription Factors Family and Kinases Regulating Lamina Joint Development

**Figure 6:**
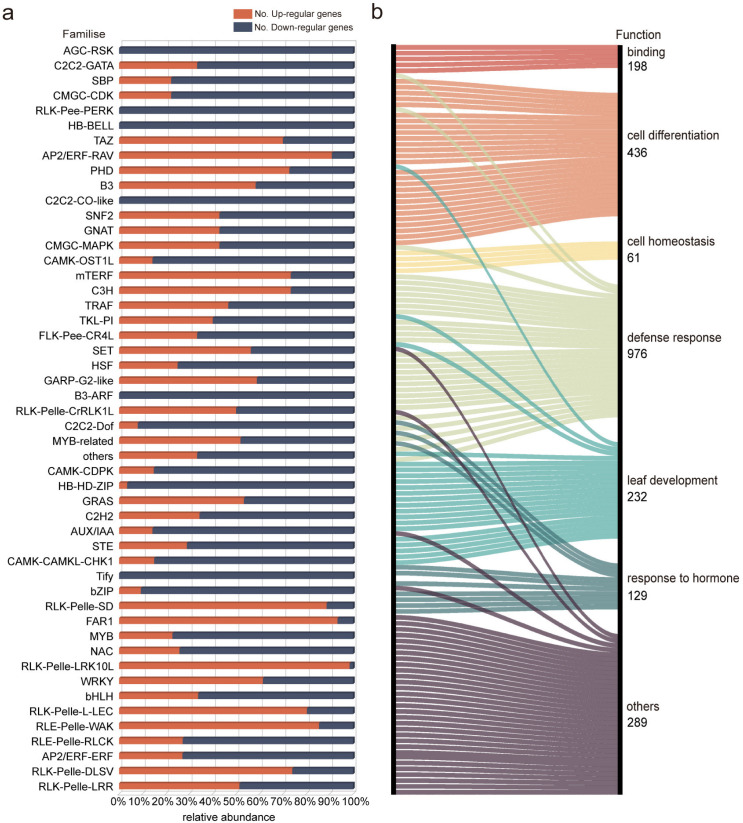
Expression profiles of DEGs encoding TFs and kinases during lamina joint development. (**a**) The top 50 TFs and kinase factors are displayed. Red denotes upregulated transcription factors, while blue indicates downregulated transcription factors. The x-axis denotes relative abundance, while the y-axis represents the different families. (**b**) Parallel chart depicting the functional categories of the TFs and kinases genes analyzed through visualization. Each color corresponds to a specific functional dataset, with the number of genes contained determined by the biological functions of reported TFs or kinases.

To identify pivotal factors influencing the cellular fate transition during lamina joint development, we conducted an exhaustive analysis of 150 transcription factors (TFs) or kinases among 2099 differentially expressed genes. These factors were categorized into 115 families, with 108 being upregulated and 130 downregulated in LG-mt relative to LG-JM22. We focused on the top 50 families or kinases with the highest number of genes. Notably, AGC-RSK-2, B3-ARF, C2C2-CO-like, HB-BELL, RLK-Pelle-PERK, and Tify manifested exhibited pronounced downregulation during lamina joint development. Conversely, RLK-Pelle-LRK, FAR1, AP2/ERF-RAV, RLK-Pelle-SD, and RLK-Pelle-WAK were upregulated (Figure 6a).

The biological functional roles of 2099 genes encoding 150 TFs/kinases were thoroughly analyzed and classified into seven distinct functional categories. Among these, 198 genes were associated with binding functions, 436 with cell differentiation, 61 with cell homeostasis, 232 with leaf development, and 129 with hormone response. Furthermore, 976 genes were attributed to defense response, while the remaining 289 were categorized under other functional groups. Through data visualization, it became evident that TFs/kinases such as RLK-Pelle-LRR, RLK-Pelle-WAK, NAC, and MYB potentially play pivotal roles in the developmental processes of the lamina joint (Figure 6b; Table S2).

## 4. Discussion

Mutants displaying altered lamina joint development are valuable assets for exploring the mechanisms underlying erect leaf formation. While extensively studied in maize and rice, such mutants are relatively scarce in wheat. *cpa* is an erect leaf mutant line derived from fast neutron irradiation breeding, characterized by erect leaf morphology controlled by a single recessive gene [19]. Plants exhibit reduced lamina joint structures and upright leaf phenotype, with a leaf angle of approximately 11° for the flag leaf. The gene controlling this trait, TaSPL8, is located on chromosome 2D and encodes the SQUAMOSA PROMOTER BINDING-LIKE (SPL) protein, which participates in the signaling pathway of brassinosteroids. *mths29* shares similarities with *cpa*, as it is also a stable erect leaf mutant obtained through fast neutron irradiation mutagenesis and directional screening. However, this mutant is controlled by a single dominant gene. Its phenotype is characterized by the complete absence of the lamina joint in the inverted second and flag leaves, resulting in a phenotype where leaves grow tightly against the stem, with a leaf angle of approximately 0°. Molecular marker analysis has linked the candidate gene to chromosome 5A. At present, most of the identified erect leaf genes are located within the recessive inheritance of the second group of chromosomes, including *eli-a* and *li* in barley, *Mrs1* and *el* in rye, as well as *lg1*, *lg2*, and *lg3* in maize [19,29,30,31]. However, there is little research on QTLs or genes associated with erect leaves on the fifth group of chromosomes. Notably, In the *LM* erect leaf mutant derived from the D genome of *Aegilops tauschii*, the candidate gene *Lgt* has been localized to the short arm of chromosome 5D [20]. However, this gene is distant from the positioning region of candidate genes in *mths29*. Morphological and genetic analyses have revealed that *mths29* represents a novel line characterized by erect leaf traits, presenting the potential for enhanced yield through dense planting practices. Delving into the genetic basis of its erect leaf phenotype promises to offer a fresh perspective for studying wheat plant architecture improvement and the mechanism underlying lamina joint development.

Map-based cloning is one of the important methods to obtain target genes of crop target traits, which is suitable for crops with large genomes and complex structures such as wheat. Using BSR of the F_2_ population originating from *mths29* and Jimai22, the erect leaf QTL was initially mapped to the 478.62 Mb interval on chromosome 5A. Subsequently, a set of 215 KASP markers were designed based on polymorphic SNPs, with only 21 proving effective. Genotyping of the F_2:3_ population, coupled with field phenotype observations, further refined the mapping to a 21.16 Mb interval spanning 291.28–312.44 Mb. Subsequent analysis of the F_4_ generation pinpointed the QTL within a 12.41 Mb physical interval at 295.78–308.19 Mb. Due to its proximity to the centromere (252.5–255.5 Mb, Chinese Spring version), the recombination frequency in this region was noted to be low [32]. To achieve finer mapping of the erect leaf QTL, we established an expanded F_5_ population consisting of 5653 individual plants. Through this, we were able to localize the QTL to the 298.61–304.99 Mb interval on chromosome 5A, with a physical interval of 6.38 Mb. Subsequently, 167 non-KASP markers were developed, but only 3 were found to be effective. By utilizing 12 key recombinant individuals within the population, we further narrowed down the mapping interval to a 1.03 Mb segment. Consequently, the mapping of the erect leaf QTL in *mths29* presented significant challenges. Therefore, the gene mapping of *mths29* erect leaf is difficult.

Among the four candidate genes, the expression levels of *CG1* and *CG5* were found to be upregulated. They are implicated in protein post-translational modification, involving methylation and ubiquitination, respectively. Research indicates that methylation and ubiquitination mechanisms play crucial roles in meristem development, with their modification levels significantly impacting the normal expression of downstream genes. For instance, in Arabidopsis thaliana, mutation of JMJ14 (H3K4 demethylase) inhibits root meristem size and growth activity, leading to a shortened root phenotype [33]. Additionally, JMJ14 participates in the formation of the miP1a/b-CONSTANS (CO) complex, and ectopic methylation occurs in a JMJ14-dependent manner, potentially leading to an early flowering phenotype [34]. Protein ubiquitination serves as the initial step of the ubiquitin-proteasome degradation pathway, playing a pivotal role in programmed protein turnover and protein quality control within eukaryotic cells [35]. The down-regulated *CG21* gene encodes a 2Fe-2S iron redox protein. Currently, DGAT3 stands as the sole identified 2Fe-2S protein implicated in the biosynthesis of triacylglycerol (TAG) in plants [36]. The content of TAG influences root meristem activity and leaf growth and development [37]. Another down-regulated gene, *CG23*, encodes a protein containing the R3H domain, which modulates RNA binding ability and catalytic function [38]. Therefore, the role of the four candidate genes in the erect leaf formation in *mths29* will be studied in the future.

**Figure 7:**
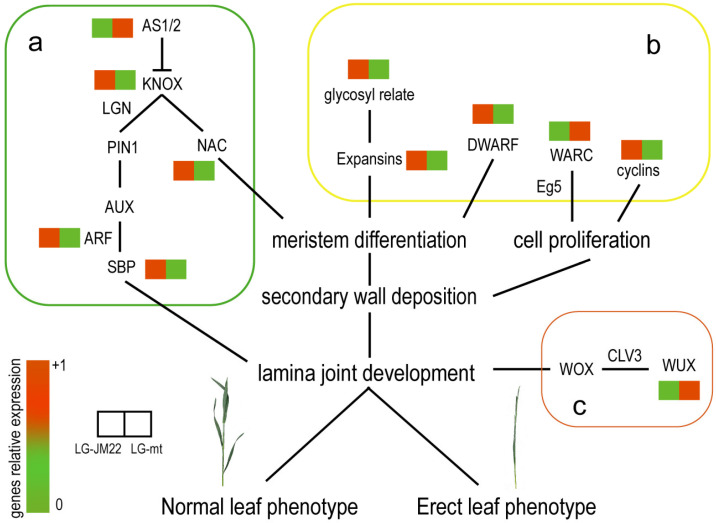
Diagram of the mechanism of erect leaf formation. Through transcriptome analysis of differentially expressed genes reported to affect erect leaf formation, the mechanism of *mths29* erect leaf formation can be divided into three parts. (**a**) centers around KNOX, influencing the formation of lamina joints through plant hormone pathways and NAC transcription factors. (**b**) involves genes related to cell division and proliferation, which disrupts the normal cell cycle process, affects cell activity balance, and decreases the mechanical strength of secondary walls such as cell walls and microtubules, resulting in the abnormal development of lamina joints. (**c**) WUX, which is specifically expressed in the apical region of leaf meristem tissue, can affect WOX transcription factors through CLV3, alter the structure of the lamina joint cell population, and produce an erect leaf phenotype.

Cell activity significantly influences lamina joint development. Research indicates that the lamina joint of *mths29* exhibits cytological features, including a reduction in the number of epidermal cells and the number of cell layers between the proximal and distal axes [39]. Therefore, this study delves into the differential expression of genes involved in boundary cells at the initiation site of preligule development, cell division, and proliferation between LG-JM22 and LG-mt. It was found that genes belonging to families such as KNOX, WUSCHEL, SBP, AS, Cyclins, and Expansins were all downregulated in LG-mt. KNOX family members are pivotal in initiating leaf primordium and ensuring the proper establishment of the leaf apical axis [9,40,41]. Its sustained expression facilitates the rapid development of leaf primordium, but it is inhibited by AS1 and AS2 during subsequent lamina joint development, leading to gene silencing [42]. WUSCHEL (WUS) exhibits specific expression in the apical region of the leaf meristem [43]. Upon transmission to the nucleus via the CLV3 signal, it induces downregulation of transcription of WUSCHEL-related homeobox (WOX) and HAIRY MERISTEM (HAM) transcription factor family members, thereby influencing the formation of the preligule zone [44]. *LG1*, belonging to the SBP family, interacts with *LG2*. It has been established that *LG1* and *LG2* play crucial roles in lamina joint development in maize, rice, and barley. Notably, maize *lg1-R* and *lg2-R* mutants demonstrate leaf age dependence, with the lamina joint absent at the seedling stage but consistent with the wild type in mature plants [14,16,30]. However, in rice and barley, the deletion of *LG1* results in a complete loss of the lamina joint throughout all developmental stages, while *oslg2* mutants do not exhibit lamina joint defects. Hence, further exploration of the mechanisms involving *LG1* and *LG2* is warranted [45,46]. Cyclins are a group of proteins closely associated with cell cycle progression, ensuring the orderly completion of DNA replication, preparation for division, actual division, and final cell division [47]. Expansin proteins, on the other hand, are known to disrupt plant cell wall structure. By breaking down hydrogen bonds within cell walls, expansins reduce wall rigidity, allowing cells to expand without rupturing [48]. These findings suggest that the erect leaf phenotype of *mths29* is influenced by cellular activity, which, in turn, is regulated by various factors, including plant hormones, ribosomes, and environmental conditions (Figure 7). Therefore, a more comprehensive investigation is needed to elucidate the mechanism underlying erect leaf formation in *mths29*.

## 5. Conclusions

In this study, we conducted QTL mapping and fine mapping of the erect leaf trait in *mths29* using an F_2_ to F_5_ population followed by recombinant analysis. Fine mapping led to the narrowing down of the target QTL to a 1.03 Mb region on chromosome 5A. Transcriptomic analysis identified four candidate genes potentially associated with the erect leaf phenotype in *mths29*. Fine mapping provides a foundation for the subsequent cloning of candidate genes responsible for the erect leaf trait in *mths29*. Subsequently, transgenic approaches and gene silencing techniques will be employed to validate the functionality of these candidate genes and to elucidate the molecular mechanisms underlying plant architecture development, facilitating crop architecture optimization. 

## Figures and Tables

**Table 1:** Segregation ratio of F_2_ population by chi-square test.

Phenotype	Actual Value	Expected Value	χ^2 a^	*p*-Value (df = 1) ^b^
Erect leaf	1310	1323	0.51	0.47
Normal leaf	454	441		
all	1764			

^a^ Calculated chi-square (χ^2^). ^b^ Likelihood that the observed segregation ratio does fit a 3:1.

## Data Availability

Data available on request.

## Supplementary Materials

The following supporting information can be downloaded at: https://www.mdpi.com/article/10.3390/biology13060430/s1, Figure S1: (a–h) Eight genes were randomly selected from the transcriptome data for quantitative PCR validation. Error bars indicate the mean ± standard error from three biological replicates. Statistically significant differences were determined by the two-tailed Student’s *t*-test. * *p* < 0.05. Table S1: The list of primers used in this study. Table S2: Transcription factors or kinase information contained in various biological functions.
