# Supplementary material for: Genetic Analysis and Fine Mapping of QTL for the Erect Leaf in Mutant mths29 Induced through Fast Neutron in Wheat
Source: Biology (Basel). 2024 Jun 11;13(6):430. doi: 10.3390/biology13060430 (PMC11201221; doi:10.3390/biology13060430)
Supplement: Supplementary file 1 [file biology-13-00430-s001.zip › Supplementary Figure S1.pdf]

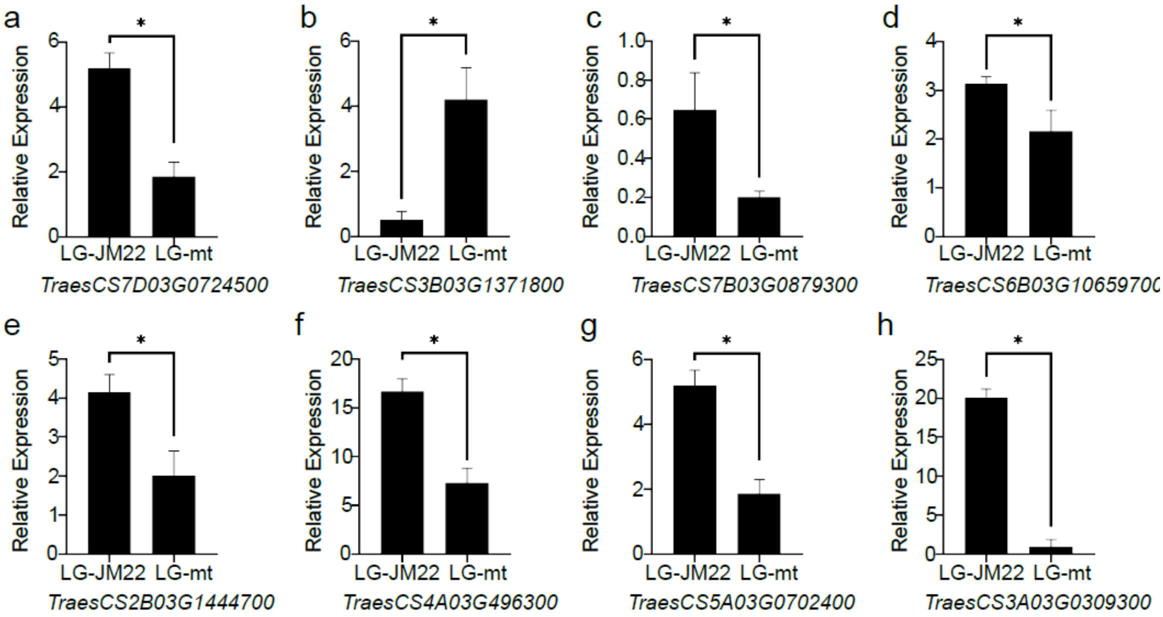

Figure S1: (a–h) Eight genes were randomly selected from the transcriptome data for quantitative PCR validation. Error bars indicate the mean ± standard error from three biological replicates. Statistically significant differences were determined by the two-tailed Student's t-test. * $p < 0.05$.
